# Supplementary material for: The mechanistic insight of a specific interaction between 15d-Prostaglandin-J2 and eIF4A suggests an evolutionary conserved role across species
Source: Biol Open. 2018 Oct 3;7(11):bio035402. doi: 10.1242/bio.035402 (PMC6262856; doi:10.1242/bio.035402)
Supplement: Supplementary information [file biolopen-7-035402-s1.pdf]

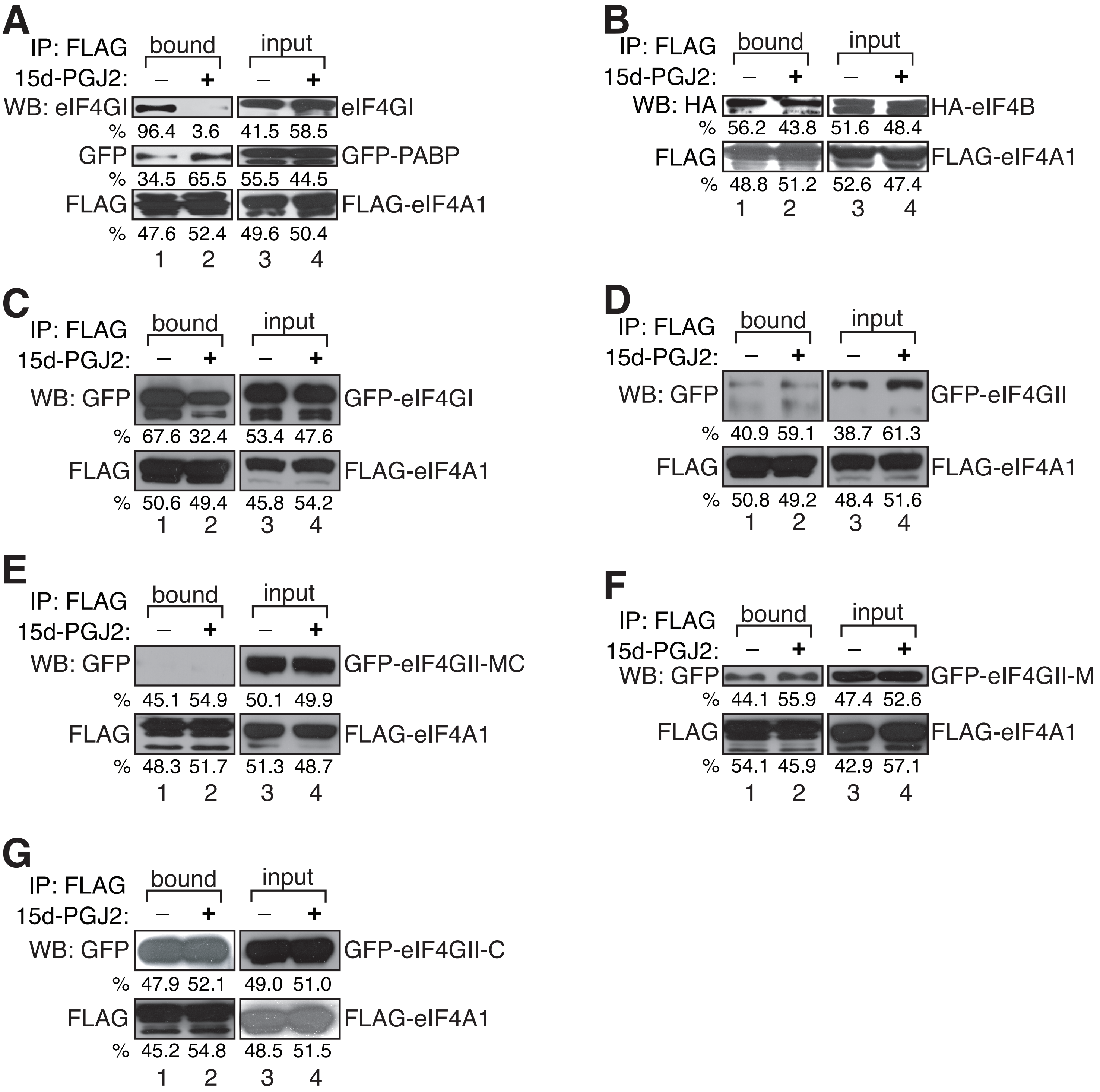
A
IP: FLAG bound input
15d-PGJ2: – + – +
WB: eIF4GI eIF4GI
% 96.4 3.6 41.5 58.5
GFP GFP-PABP
% 34.5 65.5 55.5 44.5
FLAG FLAG-eIF4A1
% 47.6 52.4 49.6 50.4
1 2 3 4
B
IP: FLAG bound input
15d-PGJ2: – + – +
WB: HA HA-eIF4B
% 56.2 43.8 51.6 48.4
FLAG FLAG-eIF4A1
% 48.8 51.2 52.6 47.4
1 2 3 4
C
IP: FLAG bound input
15d-PGJ2: – + – +
WB: GFP GFP-eIF4GI
% 67.6 32.4 53.4 47.6
FLAG FLAG-eIF4A1
% 50.6 49.4 45.8 54.2
1 2 3 4
D
IP: FLAG bound input
15d-PGJ2: – + – +
WB: GFP GFP-eIF4GII
% 40.9 59.1 38.7 61.3
FLAG FLAG-eIF4A1
% 50.8 49.2 48.4 51.6
1 2 3 4
E
IP: FLAG bound input
15d-PGJ2: – + – +
WB: GFP GFP-eIF4GII-MC
% 45.1 54.9 50.1 49.9
FLAG FLAG-eIF4A1
% 48.3 51.7 51.3 48.7
1 2 3 4
F
IP: FLAG bound input
15d-PGJ2: – + – +
WB: GFP GFP-eIF4GII-M
% 44.1 55.9 47.4 52.6
FLAG FLAG-eIF4A1
% 54.1 45.9 42.9 57.1
1 2 3 4
G
IP: FLAG bound input
15d-PGJ2: – + – +
WB: GFP GFP-eIF4GII-C
% 47.9 52.1 49.0 51.0
FLAG FLAG-eIF4A1
% 45.2 54.8 48.5 51.5
1 2 3 4

Supplement Figure 1. The effect of 15d-PGJ2 treatments on the interactions between translation initiation factors. (A) and (B) We have adopted this data from previous publication and reanalyzed it using ImageJ for quantification (6). (C)-(G) 293T cells were co-transfected with GFP-eIF4GI full length (C), GFP-eIF4GII full length (D), GFP-eIF4GII-MC (E), GFP-eIF4GII-M (F), and GFP-eIF4GII-C (G) and FLAG-eIF4A1. Cells were lysed then treated with EtOH or 10 μM of 15d-PGJ2 at 30°C for 1 hour. Immunoprecipitation was performed as described above then Western-blot analysis was performed with anti-FLAG and anti-GFP antibodies.

Biology Open • Supplementary information

A

```
eIF4A-h    MSASQDSRSRDNGPDGMEPEGVIESNWNEIVDSFDDMNLSESLLRGIYAY 50
MjDEAD     ----------------MEVE-----------YMNFNELNLSDNILNAIRNK 24
                           ** *            .*:::***:.:*..*

eIF4A-h    GFEKPSAIQQRAILPCIKG-YDVIAQAQSGTGKTATFAISILQQIELDLK 99
MjDEAD     GFEKPTDIQMKVIPLFLNDEYNIVAQARTGSGKTASFAIPLIELVN-ENN 73
           *****: ** :.*    ::. *:::***::*:****:***.:::  :: : :

eIF4A-h    ATQALVLAPTRELAQQIQKVVMALGDYMGASCHACIGGTNVRAEVQKLQM 149
MjDEAD     GIEAIILTPTRELAIQVADEIESLKGNKNLKIAKIYGGKAIYPQIKALKN 123
           . :*::*:****** *:  . : :*  .   .     **. :  .::: *:

eIF4A-h    EAPHIIVGTPGRVFDMLNRRYLSPKYIKMFVLDEADEMLSRGFKDQIYDI 199
MjDEAD     AN--IVVGTPGRILDHINRGTLNLKNVKYFILDEADEMLNMGFIKDVEKI 171
               *:******::* :**  *. * :* *:*********. ** .:: .*

eIF4A-h    FQKLNSNTQVVLLSATMPSDVLEVTKKFMRDPIRILVKKEELTLEGIRQF 249
MjDEAD     LNACNKDKRILLFSATMPREILNLAKKYMGDYSFIKAKIN----ANIEQS 217
           ::  *.:.:::*:***** ::*:::**:* *   * .* :      .*.*

eIF4A-h    YINVEREEWKLDTLCDLYETLTITQAVIFINTRRKVDWLTEKMHARDFTV 299
MjDEAD     YVEVN-ENERFEALCRLLKNKEFYG-LVFCKTKRDTKELASMLRDIGFKA 265
           *::*: *: ::::** * :.  :   ::* :*:*... *:. ::  .*..

eIF4A-h    SAMHGDMDQKERDVIMREFRSGSSRVLITTDLLARGIDVQQVSLVINYDL 349
MjDEAD     GAIHGDLSQSQREKVIRLFKQKKIRILIATDVMSRGIDVNDLNCVINYHL 315
           .*:***:.*.:*: ::* *:. . *:**:**::*****:::. ****.*

eIF4A-h    PTNRENYIHRIGRGGRFGRKGVAINMVTEEDKRTLRDIETFYNTSIEEMP 399
MjDEAD     PQNPESYMHRIGRTGRAGKKGKAISIINRREYKKLRYIERAMKLKIKKLK 365
           * * *.*:***** ** *:** **.::...: :.** **   : .*:::

eIF4A-h    LNVADLI 406
MjDEAD     FG----- 367
           :.
```

B

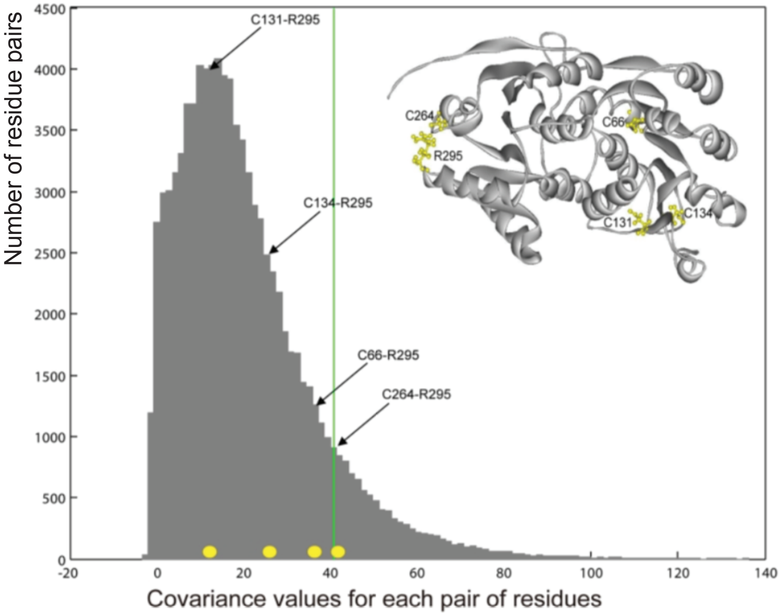

C

```
Vertebrate
  gi|4503529|Human          LDTLCDLYETLTITQAVIFINTRRKVDWLTEKMHARDFTVS
  gi|40786436|Rat           LDTLCDLYETLTITQAVIFINTRRKVDWLTEKMHARDFTVS
  gi|114666180|Chimpanzee   LDTLCDLYETLTITQAVIFINTRRKVDWLTEKMHARDFTVS
  gi|21450625|Mouse         LDTLCDLYETLTITQAVIFINTRRKVDWLTEKMHARDFTVS
  gi|77735407|Cow           LDTLCDLYETLTITQAVIFINTRRKVDWLTEKMHARDFTVS
  gi|58331980|Frog          LDTLCDLYETLTITQAVIFINTRRKVDWLTEKMHARDFTVS
Invertebrate
  gi|17136248|Drosophila    LGTLCDLYDTLSITQSVIFCNTRRKVDQLTQEMSIHNFTVS
  gi|15221761|Arabidopsis   LETLCDLYETLAITQSVIFVNTRRKVDWLTDKMRSRDHTVS
  gi|71987143|Worm          FDCLCDLYNVVNVTQAVIFCNTRRKVDTLTEKMTENQFTVS
  gi|19115766|Fission Yeast LDTLCDLYETVTVTQAVIFCNTRRKVDWLTEQLTERDFTVS
  gi|6322912|Budding Yeast  YECLTDLYDSISVTQAVIFCNTRRKVEELTTKLRNDKFTVS
```

Supporting Figure 2. Covariance and conservation of C-R pair. (A) The sequence alignment of human eIF4A-1 with MjDEAD. The conserved motifs of the DEAD box helicase are highlighted with gray boxes. (B) Covariance value for each pair of amino acid residues within eIF4A. The histogram shows the number of residue pairs corresponding to each covariance value. The green line represents the covariance value of top 10 percentile. The covariance values for C-R pairs are highlighted with yellow dots. The four C-R pairs are presented in the bracket. In the figure inside, four cysteins (C66, C131, C134, C264) and R295 are colored in yellow. (C) Conserved C-R pair in vertebrate orthologues of human eIF4A-1. The 11 orthologues of human eIF4A-1 from vertebrate and invertebrate are aligned. Cys and Arg are boxed in gray in alignment of 11 orthologues of human eIF4A-1.

Biology Open (2018): doi:10.1242/bio.035402: Supplementary information

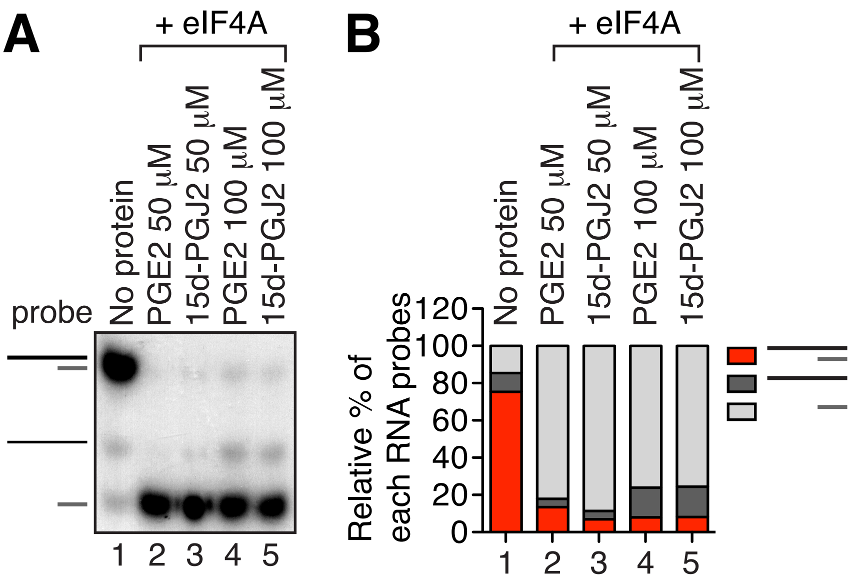

Supplement Figure 3. The effect of C264/R295 mutation on the 15d-PGJ2 binding to eIF4A. (A) Helicase assay was performed using purified His-eIF4A and radiolabeled oligonucleotides in the presence of PGE2 or 15d-PGJ2. (B) The gel images of (A) were analyzed with ImageJ and the relative amount of double strand, longer primer, and shorter primer was calculated.

Biology Open • Supplementary information

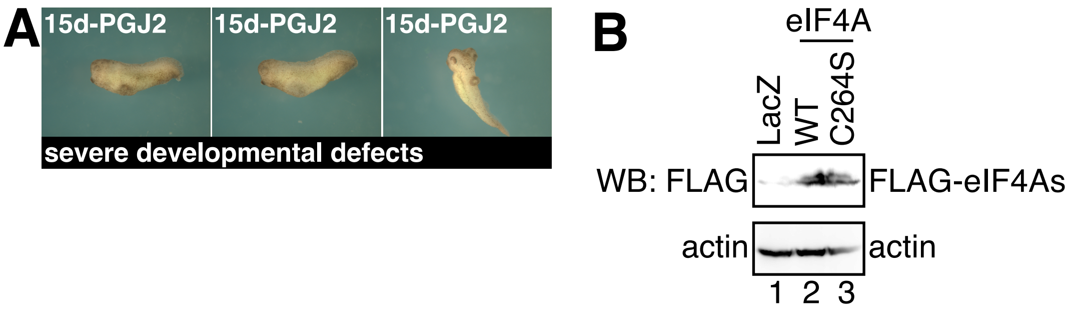

C

```
>Sf9LR650001-5-1-C796
          Length = 2231

 Score =  617 bits (1592), Expect = e-177
 Identities = 306/400 (76%), Positives = 347/400 (86%)
 Frame = -3

Query: 4    RNEIPQDGPASMEPEGVIESTWHEVYDNFDDMNLREELLRGIYGYGFEKPSAIQQRAIIP 63
            R +   DGP  MEP+G +++ WH+V ++FDDMNL+EELLRGIY YGFEKPSAIQQRAI+P
Sbjct: 2115 RIKATMDGPPGMEPQGALDTNWHQVVESFDDMNLKEELLRGIYAYGFEKPSAIQQRAIMP 1936

Query: 64   CVRGRDVIAQAQSGTGKTATFSIAILQQIDTSIRECQALILAPTRELATQIQRVVMALGE 123
            C++GRDVIAQAQSGTGKTATFSI+ILQQIDTSIRECQALILAPTRELA QIQ+VV+ALG+
Sbjct: 1935 CIQGRDVIAQAQSGTGKTATFSISILQQIDTSIRECQALILAPTRELAQQIQKVVIALGD 1756

Query: 124  YMKVHSHACIGGTNVREDARILESGCHVVVGTPGRVYDMINRKVLRTQYIKLFVLDEADE 183
            ++    HACIGGTNVRED R LESG HVVVGTPGRVYDMI R+ LR   IKLFVLDEADE
Sbjct: 1755 HLNAKCHACIGGTNVREDVRQLESGVHVVVGTPGRVYDMITRRALRANTIKLFVLDEADE 1576

Query: 184  MLSRGFKDQIQDVFKMLPPDVQVILLSATMPPDVLEVSRCFMRDPVSILVKKEELTLEGI 243
            MLSRGFKDQI DVFKML  DVQVILLSATMP DVLEVSRCFMRDPV ILV+KEELTLEGI
Sbjct: 1575 MLSRGFKDQIHDVFKMLSADVQVILLSATMPDDVLEVSRCFMRDPVRILVQKEELTLEGI 1396

Query: 244  KQFYVNVKQENWKLGTLCDLYDTLSITQSVIFCNTRRKVDQLTQEMSIHNFTVSAMHGDM 303
            KQFY++++ E WKL TLCDLYDTLSI Q+VIFCNTRRKVD LT+ M   +FTVSAMHGDM
Sbjct: 1395 KQFYISIELEEWKLETLCDLYDTLSIAQAVIFCNTRRKVDWLTESMHERDFTVSAMHGDM 1216

Query: 304  EQRDREVIMKQFRSGSSRVLITTDLLARGIDVQQVSLVINYDLPSNRENYIHXXXXXXXX 363
            +QR+REVIM+QFR+GSSRVLITTDLLARGIDVQQVS VINYDLP+NRENYIH
Sbjct: 1215 DQREREVIMRQFRTGSSRVLITTDLLARGIDVQQVSCVINYDLPTNRENYIHRIGRGGRF 1036

Query: 364  XXXXVAINFITDDDRRILKDIEQFYHTTIEEMPANIADLI 403
                +AINF+T+ DRR LKDIE+FYHT+I EMP+++A+LI
Sbjct: 1035 GRKGIAINFVTEADRRALKDIEEFYHTSISEMPSDVANLI 916
```

Supporting Figure 4. Continued data of the effect of 15d-PGJ2 on various species. (A) Examples of severe developmental defects of *Xenopus* by 15d-PGJ2 treatment. (B) The expression of FLAG-eIF4A mRNA used in Fig. 4E experiments were confirmed by western blot analysis. (C) Amino acid sequence of *Spodoptera frugiperda* eIF4A. Reference sequence was human eIF4A and the region contains C264~R295 is highlighted.

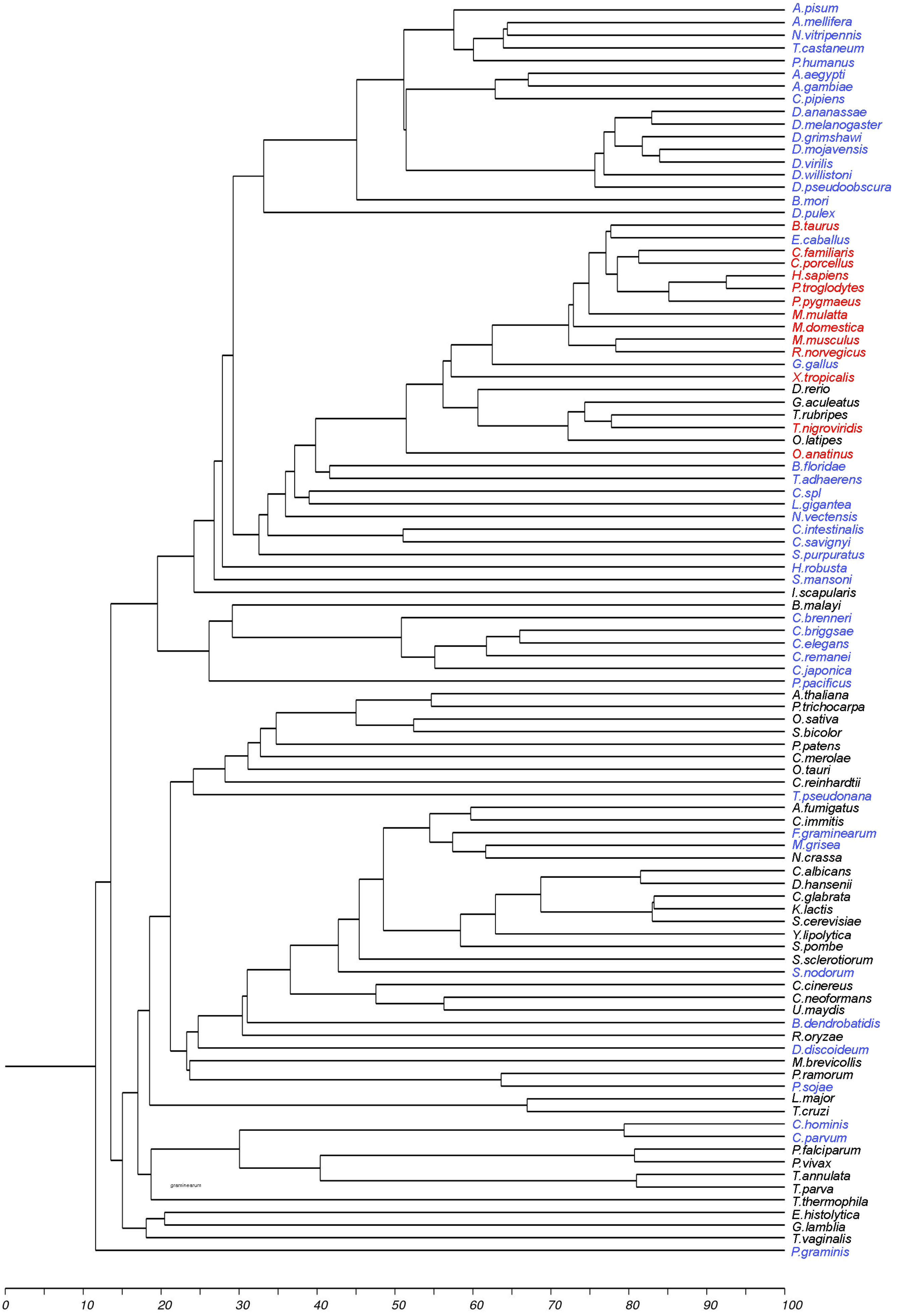

Biology Open • Supplementary information

Supplement Figure 5. Orthophylogram of PGDS based on the average fraction of InParanoid orthologues between species (57)**.** Blue/Red and black represent the species with or without the orthologues of human PGDS, respectively. Red represents the species that the orthologues of both PGDSs, HPGDS (entrezID: 27306) and LPGDS (entrezID: 5730), are found. Blue represents the species that the orthologues of HPGDS (entrezID: 27306) are found. Black represents the species that had no orthologues of PGDSs. (B) The sequence alignment of human eIF4A-1 with MjDEAD. The conserved motifs of the DEAD box helicase are highlighted with gray boxes.

Biology Open • Supplementary information
